# Gamification Approach to Provide Support About the Deferral Experience in Blood Donation: Design and Feasibility Study

**DOI:** 10.2196/50086

**Published:** 2024-06-14

**Authors:** Roberto Espinoza Chamorro, Luciano H O Santos, Yukiko Mori, Chang Liu, Goshiro Yamamoto, Tomohiro Kuroda

**Affiliations:** 1 Graduate School of Informatics Kyoto University Kyoto Japan; 2 Fitting Cloud Inc. Kyoto Japan; 3 Kyoto University Hospital Kyoto Japan

**Keywords:** blood donation, deferral experience, Theory of Planned Behavior, Self-Determination Theory, gamification, ICT design, motivation, patient education, prototype, feasibility

## Abstract

**Background:**

Multiple studies have examined the impact of deferral on the motivation of prospective blood donors, proposing various policies and strategies to support individuals who undergo this experience. However, existing information and communications technology systems focused on blood donation have not yet integrated these ideas or provided options to assist with the deferral experience.

**Objective:**

This study aims to propose an initial gamified design aimed at mitigating the impact of the deferral experience by addressing the drivers of awareness and knowledge, interaction and validation, and motivation. Additionally, the study explores the feasibility of implementing such a system for potential users.

**Methods:**

We conducted a literature review focusing on the dynamics of motivation and intention related to blood donation, as well as the deferral situation and its impact on citizens. Through this review, we identified weak donor identity, lack of knowledge, and reduced motivation as key factors requiring support from appropriate interventions. These factors were then defined as our key drivers. Taking these into account, we proposed a gamification approach that incorporates concepts from the MDA framework. The aim is to stimulate the aforementioned drivers and expand the concept of contribution and identity in blood donation. For a preliminary evaluation, we designed a prototype to collect feedback on usability, usefulness, and interest regarding a potential implementation of our proposed gamification approach.

**Results:**

Among the participants, a total of 11 citizens interacted with the app and provided feedback through our survey. They indicated that interacting with the app was relatively easy, with an average score of 4.13 out of 5 when considering the 11 tasks of interaction. The SUS results yielded a final average score of 70.91 from the participants’ answers. Positive responses were received when participants were asked about liking the concept of the app (3.82), being likely to download it (3.55), and being likely to recommend it to others (3.64). Participants expressed positivity about the implementation of the design but also highlighted current shortcomings and suggested possible improvements in both functionality and usability.

**Conclusions:**

Although deferral is a common issue in blood donation, there is a missed opportunity in existing ICT services regarding how to effectively handle such experiences. Our proposed design and implementation seem to have captured the interest of prospective users due to its perceived positive usefulness and potential. However, further confirmation is needed. Improving the design of activities that currently rely heavily on extrinsic motivation elements and integrating more social components to create an enhanced activity loop for intrinsic motivation could further increase the value of the proposed project. Future research could involve conducting a more specialized and longitudinal design evaluation with a larger sample size.

## Introduction

### Blood Donation and Deferral Experience

Blood donation is an altruistic, socially responsible, and even a self-care activity. However, the commitment required to participate can be deterred by uncomfortable experiences, negatively affecting motivation to donate [1]. Preventing such experiences and reducing their impact could preserve the intention and willingness of citizens to continue donating blood.

One significant deterrent is the deferral experience [2], which means being disqualified from donating blood based on eligibility criteria. It is especially impactful in young and first-time donors, often resulting in abandonment [3]. Negative emotions are commonly reported during the deferral process [4]. Such negative experiences can deter potential donors when shared with their social circles [5]. Similarly, studies consistently identify negative interactions with staff, feelings of rejection, and confusion about deferral reasons as primary factors reducing return intention and motivation [6]. Some deferred donors misunderstand their deferral conditions, erroneously believing they cannot donate for longer periods or even permanently [7]. Communication and information gaps contribute to these misconceptions [8].

### Challenges and Potential Solutions

To counter deferral effects, strategies such as enhanced communication, clear deferral information, and targeted recruitment show promise [4]. However, these solutions require substantial planning and resources, often unavailable to many blood centers. In that regard, information and communications technology (ICT) platforms, inspired by successful implementations in health promotion and telemedicine [9,10], could facilitate such support. As existing apps focus mostly on the donation itself and on supporting citizens for their next donation [11,12], there is an opportunity to offer unique value by tailoring systems to address the deferral experience. However, to that end, understanding the psychological responses and specific needs of deferred donors is crucial. Temporary deferrals necessitate motivation, health improvement, and eligibility [13], whereas permanently deferred donors (those unable to donate anymore) could still contribute indirectly through activities such as promoting donation.

Regarding the motivation topic, an approach that has gained notoriety is gamification, which involves the use of game-design elements in nongaming contexts [14]. Some blood donation centers worldwide use gamification, rewarding donors with badges, gifts, and certificates [15]. Furthermore, government blood donation apps in countries such as the United States and Canada have integrated gamified elements into their ICT services, aiming to boost donor motivation [16,17]. Although the impact on blood donation is yet to be studied, gamification has proven effective in therapy commitment and health self-monitoring [18,19]. Considering this, gamification holds promise for increasing motivation in the blood donation context.

However, motivation is not the only factor to consider for a possible proposal. Previous studies have identified various deterrents, stemming from deferrals, health conditions, or environmental factors, which influence citizens’ future intentions and behaviors [4]. In this study, we reviewed previous findings, as well as the results of a preliminary survey, to identify pertinent topics and form the foundation of our proposed design for an ICT system that aims to support (also) deferred donors.

### Theory of Planned Behavior and Extensions in Blood Donation

The Theory of Planned Behavior (TPB) [20], an extension of the Theory of Reasoned Action, asserts that specific behavior is determined by intention, influenced by attitude, subjective norm, and perceived behavioral control. In the context of blood donation, the TPB proposed that positive evaluation of the act (attitude), social expectations (subjective norm), and belief in individual control over donation (perceived behavioral control) dictate the decision to donate.

Previous studies have found that the TPB explains between 32% and 50% of the variance in intention and 27% and 36% of the variance in behavior [21,22]. To enhance predictive power, the framework was extended due to the inconsistent link of the subjective norm [23]. In blood donation, additional constructs were incorporated based on psychological differences among nondonors, novices, and repeat donors. Moral norms, descriptive norms, past behavior, and self-identity were included as predictors [20,24,25].

Systematic reviews have shown that self-efficacy, donor identity, and anticipated regret have medium positive effects on both intention and behavior. Conversely, deferral has a medium negative impact, leading to a decrease in subsequent donations among experienced donors [26,27]. Past behavior or habit explains 19% additional variance in blood donation behavior for those donating 5 times or more [28]. Habit, suggested to be context bound, is viewed as an external motivator, whereas self-identity, which pertains to one’s role in society as a blood donor, is defined as an internal motivator [29-31]. Both habit and self-identity significantly influence repeat blood donation behavior, with past behavior likely forming identity [23].

### Self-Determination Theory and Motivation and Gamification

As the TPB applies mostly to situational-level intentions [32], blood donation studies primarily rooted in the TPB have expanded their scope to incorporate Self-Determination Theory (SDT) in the last decade [33,34]. SDT, a theory of human behavior and personality development, emphasizes social-contextual factors supporting human growth through satisfying basic psychological needs for *competence* (effectiveness of my actions in my current environment), *relatedness* (social involvement and relation with others), and *autonomy* (internal need to be responsible for your own meaningful choices) [35]. It proposes that internally motivated behaviors persist, while external motivations can become internalized under appropriate socioenvironmental conditions [36].

SDT categorizes behavior on a continuum from amotivation (nonregulated behavior) to extrinsic motivation (external to integrated regulation) to intrinsic motivation (intrinsic regulation). Extrinsic motivation refers to acting in a certain way or doing a specific action because it leads to a separable outcome or reward. By contrast, intrinsic motivation refers to acting in a certain way or doing a specific action because the act itself is inherently satisfying. Integrating the TPB and SDT, studies have revealed that SDT’s motivational orientations explained an additional 14% of the variance in blood donation intention compared with TPB-only models [37]. Amotivation had a negative direct effect on intention, while external motivation had no overall effect on intention but a positive effect on amotivation [38]. By contrast, introjected regulation had positive direct and indirect effects on intention, and autonomous motivation predicted intention directly and via attitudes, subjective norms, and perceived behavioral control [33,38].

### Gamification Concepts and Frameworks

As SDT discusses the impact of motivation on behavior, it was considered the foundation for implementing gamification, as it does not aim to directly affect an outcome, but to change a target behavior (by affecting psychological factors) that can lead to that outcome [39,40]. To achieve this, the system can utilize its various design components, as outlined by the Mechanics, Dynamics, and Aesthetics Framework (MDA) [41], which served as the primary reference for our study. The framework comprises mechanics, which encompasses specific game components such as data representation and algorithms; dynamics, which refers to the interactions between these mechanics and player inputs over time; and aesthetics, which aims to elicit desirable emotional responses from players when they engage with the game system. These components are integrated to drive either extrinsic or intrinsic motivation, considering the targeted changes in human behavior [42-44].

Extrinsic motivation can drive behavior but may fade without external rewards, while intrinsic motivation leads to long-term positive effects on intention and behavior [45,46]. Thus, most gamified approaches recommend prioritizing intrinsic motivation in the design process. In that regard, users can be categorized according to the recognized characteristics and that drives them in the gamified implementations [47]: socializers (motivated by *relatedness*), free spirits (motivated by *autonomy*), achievers (motivated by *competence*), philanthropists (motivated by *purpose and meaning*), players (motivated by *rewards*), and disruptors (motivated by *change*). The players and disruptors categories can be further divided according to their behavior.

Considering the previous concepts and relationships of gamification and SDT, DiTommaso and Taylor [39] defined a framework in which they propose the following steps for design: discover the reason to gamify, identify players’ profiles and motivational drivers, set up goals and objectives, describe skills and desired outcomes, and playtest among others. Another design framework with similar foundations is the Six Steps to Gamification [48], which also takes influence from the MDA. It proposes the following steps: definition of business objectives, target expected behavior, description of players, design of activity loops, do not forget the fun, and deploy appropriate tools. Although not domain specific, these adaptable frameworks can guide gamification projects and were also used for reference in our study.

### Deferral Experience and Effects in Return Rate

From the literature review, we chose to focus on recurrent and impactful issues related to the deferral experience, especially the ones that aligned with the constructs from the TPB and SDT. For example, the construct of self-identity (blood donor identity in this case) from the extended TPB can be associated with the negative feelings from a deferral. More specifically, a deferral, which can generate a feeling of rejection in the unsuccessful participant [13], can threaten the citizen’s self-perception as a capable blood donor (identity), as the inability to participate diminishes their possibility of building experiences and forming a habit (especially in the cases of new and young donors). Similarly, confusion and misunderstandings in deferral make a successful blood donation seem more complex and difficult than it is, affecting citizens’ perceived behavioral control (TPB construct). As indicated by Gemelli et al [1] and Hillgrove et al [13] negative experiences can reduce the motivation for future involvement, particularly for long-term or permanently deferred donors, eroding their sense of self-efficacy.

To further explore the relationship between the deferral experience and intention, we also took into account the findings of a preliminary survey involving Japanese citizens [49], in which a total of 208 participants were recruited. In the survey, the dependent variable was “Intention to donate again after deferral” (a 6-point Likert scale question with the values 1=not anymore, 2=not for a while, 3=I don’t know, 4=maybe, after a while, 5=yes, unless rejected again, and 6=yes, I would). Citizens were asked whether they heard or knew about the concept of deferral and whether they had experienced a deferral case, as well as their future intention in a possible deferral scenario. The results implied a possible relation between deferral and reduced intention to donate (following previous studies). However, the data also suggested a positive relation between preventive awareness of the deferral experience and intention to donate. Donors and nondonors who had knowledge about the deferral concept indicated higher intention of future participation even after a possible deferral scenario.

### Objectives

Considering the literature review, we focused on recurrent issues that could be addressed with a gamification approach, taking into account the connections between the deferral experience, their issues, and motivation. The topics we chose were as follows:

- Lack of knowledge about deferral: Some of the negative feelings appear because citizens are not knowledgeable of the topic, are not retaining the information, or have misunderstood it.
- Weak donor identity: citizens feeling rejected and lacking validation.
- Reduced motivation: citizens losing interest in addressing the deferral reason or losing interest in contributing to the future.

Additionally, considering the evaluated strategies to mitigate the negative impact of deferral from the analyzed literature [4,50], we defined the main drivers for our approach. First, to provide awareness and knowledge about deferral by making learning interesting to the citizens (*awareness and knowledge*). Next, to increase the scenarios of interaction and validation for deferred donors to nurture their identity (*interaction and validation*). Lastly, to provide motivational drivers for deferred donors to regularly engage in activities related to blood donation (*motivation*).

After that, we worked on the design of activities that could be implemented with the gamification framework while targeting the drivers selected regarding the deferral experience. After completing the initial design, we implemented a prototype with basic features and integration for a feasibility study, collecting feedback about the usability and receptivity of potential users to discuss the future value of the idea of offering a service regarding the deferral experience, our proposed design, and its implementation.

For this study, we explored the following research questions (RQs):

- RQ1: Will our gamified design that focuses on the previously mentioned drivers with regard to the deferral experience in blood donation have a positive reception from potential users?
- RQ2: Will our initial prototype implementation of the design be considered usable and useful in its current iteration?

## Methods

### Conceptualizing a Gamification Approach for the Deferral Experience

#### Overview

In this study, we are adopting an approach similar to the gamification frameworks mentioned previously [40,41,48], while also taking into account the unique requirements of individuals in blood donation. We have adapted the steps and elements of these frameworks to provide support specifically addressing the deferral experience and focusing on the main drivers mentioned.

#### Definition of Approach Objectives

We redefined our target users to include not only deferred donors but also regular donors and potential donors who might face deferral in the future. Our focus broadened to cater to anyone interested in the topic, aligning with our objective of providing deferral support. We concentrated on 3 main issues: lack of knowledge about deferral, weak donor identity, and reduced motivation, translating these into drivers for our gamification approach: *awareness* *and knowledge*, *interaction and validation*, and *motivation*.

#### Target Expected Behavior

The next step was to define the citizens’ expected behavior when interacting with our proposed gamification implementation. For our approach, we wanted the design to nurture the drivers, and as a consequence, possibly affect future intentions.

For *awareness and knowledge*, we expected users to engage in educational activities that both teach them about and test their understanding of the deferral experience and strategies for improvement. For *interaction and validation,* we expected users to get involved in discussions, in sharing experiences, and in supporting one another, improving the sense of community. In terms of *motivation*, our goal was to encourage users to access the system regularly, ideally once or twice per week, considering the prolonged pace between blood donations.

#### Description of Users

For our target group, while we initially expected to focus on the deferred donors, the results from the preliminary survey guided us to design the service as a preemptive one (including regular donors and nondonors), to nurture the identity of the users and prepare them against a deferral scenario. Designed primarily for young citizens (20-30 years) yet accessible to older individuals, the approach incorporated specific design elements reflecting the regional context (Japan). However, the core of the approach was intended to be adaptable, considering possible future adaptations for other regions.

In the context of the gamification approach, considering that the potential users (citizens) would not have the same goals or motivations (following the connection with SDT), for this study, we focused on targeting the players, the socializers, the free spirits, the achievers, and the philanthropists.

#### Design of Activity Loops

##### Macrolevel Progression Loops

The gamification approach aims to motivate citizens, particularly deferred blood donors, to stay engaged with blood donation–related activities. Although encouraging future donations is the ultimate goal, maintaining interest in the topic and promoting contributions to other related areas are also crucial. The design focuses on creating macrolevel progression loops for the drivers of *awareness and knowledge*, as well as *interaction and validation.*

##### Initial Outline

User progression is represented through levels. Levels increased based on experience points earned from various activities. Points earned could be exchanged for basic title characters. Special characters are unlocked as users progress, with higher levels requiring more points for unlocking. Higher user levels unlock additional activity options, which yield more points.

For the microlevel, we first defined some basic loops for the foundation of the design. For example, one of the initial hurdles considered was that, independent of any learning or social activity that could be designed, their value would not be achieved if the users were not motivated to access the ICT system. In that regard, we considered a simple loop of providing a reward to initially push the user to use the system: if the user logs-in to the system, they receive a message about their current streak and earn some points. Users will earn more points according to how often they connect to the app and how high is their level. With regard to *awareness and knowledge*, we aimed to make the users both learn about deferral and review their current knowledge. For this purpose, the initial idea for this loop was that as users learn more, they can face harder challenges. And the more successful they are, the more complex information they will be taught. With regard to *interaction and validation*, we aimed to provide some activities in which users could interact with other users, and the more interactions and levels the user has, the more options of interaction would be available to them.

However, we needed to solidify the ideas for the microlevel. To achieve this, we opted to elaborate on the design with greater detail. We chose to do this by following the MDA framework, first from the user perspective, then transitioning to the designer perspective to finalize the activities’ design.

##### Definition of MDA Aesthetics

For the driver *awareness and knowledge*, we aimed to nurture a habit in the users of learning about deferral. For *interaction and validation*, the expected behavior was to generate regular engagement in the users. To that end, specifically for the players, we first selected *submission*, which means the design would allow users to interact with the system as a pastime. Our goal was to present a variety of activities offering rewards and collectible items to enhance user enjoyment. However, this approach may heavily rely on extrinsic motivations, potentially overshadowing the altruistic aspect of blood donation. Thus, we needed to be cautious in its implementation to avoid solely focusing on rewards. To address this, we selected *fellowship* as a social framework to appeal to users who value social experiences.

We considered possible ways to make users interact with others, possibly in cooperation or competition. *Challenge* (experience as obstacle course), *discovery* (experience as uncharted territory), and *expression* (experience as self-discovery) were also chosen as they are more related to intrinsic motivations, which we wanted to favor over the extrinsic motivation, which was aimed to be used only as the trigger for the conduct of the users.

##### Definition of MDA Dynamics

We initially drafted dynamics outlines to connect the drivers and the aesthetics. For instance, in terms of *awareness and knowledge,* users could opt to heighten the difficulty of their learning process, introducing an element of risk that could generate a *challenge.* Additionally, we explored the possibility of randomizing the information users received, with variations based on their actions within the environment, thus fostering a sense of *discovery.*

By contrast, for *interaction and validation*, we aimed for users to be able to choose the type of recognition they would get, allowing for *expression*. They should also decide what they could share with others and try to encourage them to perform certain actions, creating *fellowship*. From these initial ideas, we expanded into more detailed dynamics in the designer perspective iteration of the MDA.

##### Definition of MDA Mechanics

Generic mechanics are introduced, incorporating points, levels, and characters for onboarding. Points served as rewards for participating in different activities (the amount was adjusted per result), to create a sense of progression (the historical record was tracked to calculate the current level of the user), and to be used as a currency in the system. Levels were also used for progression. They increased according to the number of participations, providing recognition and incremental rewards. They were used as a certain multiplier in the activity rewards and to unlock new and special characters in the exchange store.

Characters were chosen as part of the representation and recognition of the users, being the main extrinsic reward of the gamification approach. However, they were integrated to appeal to both extrinsically and intrinsically motivated players, aiming to reduce the dependency on the extrinsic component. For example, with customization, they would target free spirits; if they were collectible, they would target players and achievers. As general rules, every registered user was provided with the same starting character; they could acquire more in the shop by exchanging the points they collected through the activities; they could also upgrade (defined as “evolve” inside the app) them by exchanging multiples of the same one. One character at a time could be selected to use it as their icon in their social activities, and characters would change their appearance if the user stood inactive in the system for more than a week.

Some social interaction components were included, such as a comment section and a simple feed wall for users’ posts. Both of them had an upvote or downvote mechanism for users to indicate their relevance or popularity. A certain degree of user anonymity was incorporated to reduce possible social burdens of participants when creating content. However, for regular comments, the app showed their current character (and title) and their username. The main posts were put on hold until approved by an administrator, to reduce possibly harmful or misleading content; however, regular comments did not have this restriction. These mechanics aimed to engage the socializer, the free spirit, and the philanthropist types of users.

After this first iteration, we started with the designer perspective, in which we focused on linking all the previous concepts together, defining the more specific activities available in the system for the users.

##### MDA-Based Features and Feedback Loops

Finally, we describe the design of our proposed features for the gamification approach, integrating all the previous considerations and concepts.

The first feature we defined was the “Login Reward.” Usage of blood donation apps tends to be low because of the timed nature of donating blood. However, to handle learning and engagement, as part of the onboarding, we chose to encourage users to interact with the system more often. To that end, we rewarded points if users log-in to the app regularly with up to 5 rewards per week, increasing the amount per consecutive access. We linked the reward to the level mechanic, providing additional points according to the level. Regarding the *awareness and knowledge* driver, we also included a message of advice and information regarding blood donation deferral. As dynamics, users could choose to access the app as usual or connect more times to increase their multiplier. Besides, as the level was linked to the rewards, users could choose to increase their level through other activities to receive more points. However, as users were not forced to read the advice message, we connected it with other activities to create the intended aesthetics and more complex activity loops.

The next features we defined, *quizzes* and *social poll*, were aimed to be connected with the broader activity loop and the *awareness and knowledge* driver. Quizzes have been implemented in other blood donation apps, so we included additional mechanics to make it less extrinsic, create new dynamics, and reach the intended aesthetics. We incorporated a life mechanic that resets daily, along with a difficulty level that becomes unlockable as users progress through levels, giving less incentive to guess the answer while also providing a higher risk-higher reward choice to more expert users. Additionally, feedback was provided according to the result, either congratulating the user for their right answer or guiding the user on the mistake. Furthermore, we connected the questions with the content shared through other features, so invested users will feel rewarded for learning on their own. Similar ideas were considered for the content of the *social poll*, but some mechanics that could allow for social interaction were included. Once per week, users could vote between different facts related to blood donation deferral, according to what they felt was the most interesting one. At the end of the week, users were notified of the most popular choice, and the ones who chose it were able to claim reward points. If desired, users could either discuss outside or through the app to try to get information about other users’ preferences or to coordinate a specific choice for benefit. Additionally, previous results and facts were accessible, so users could review the content and discuss it for self-learning or connection with users.

The *news sharing* feature was also connected to the previous features and the *awareness and learning* driver, as content shared on the former would be used in the latter ones. Users could like their favorite entries and could comment about them. Comments or replies from administrative users had a special identifier while regular users had the default. Administrators would try to reply to important questions, but the content of the comments or discussions was up to the users, giving them freedom for communication.

Similarly, to provide more options for the *interaction and engagement* driver, we defined the *posts* feature. Users could create posts for discussion (questions, anecdotes, suggestions, among others). If approved, the posts were shown in the app anonymously, displaying their current relevancy score. Every week, users who created new posts with high relevance would be rewarded points. While posts were defined to require approval by the administrator users, that restriction was not included in the evaluation. Posts would show in the user’s feed, by order or relevancy and created date. We aimed to reward users for meaningful content, which in itself could motivate the participation of other users in the discussions. Besides, as the more relevant ones would be highlighted in the app feed, it could provide a sense of self-worth by knowing that one’s content received a good reception from the community or that it provided value to the community, eventually motivating them to participate again in the discussions.

The next feature was “Application Alarms,” aiming to provide users with some mechanics that could support their preferences. Users had the option to enable up to 3 types of notifications: notifications when new characters were implemented, notifications for news and discussions, and a reminder of the calculated end of the deferral period. The aim was for users to voluntarily choose to get informed about their topics of interest within the app.

Finally, for this initial scope of the design, we included features that, while not creating a proper loop by themselves, were required to connect the previous features and their loops. The first one is the “Character Store,” in which users can exchange their points for available characters. The list of characters was updated in a regular schedule, with new characters being highlighted, while locked characters had a gray background. The store showed the required level, price, and current amount collected for each character. Characters being collectible were used as an extrinsic way to motivate the users to keep getting points through the other activities. By contrast, the upgrade option was linked to the title achieved by the user, which meant a special title for their effort. Users had the option to concentrate their points on either one objective or the other or to participate as much as possible to pursue both simultaneously. The other feature was the “Profile,” in which users had access to their stats (level/points), their character collection (including the upgrades and selection), and additional settings for the account.

**Figure 1:**
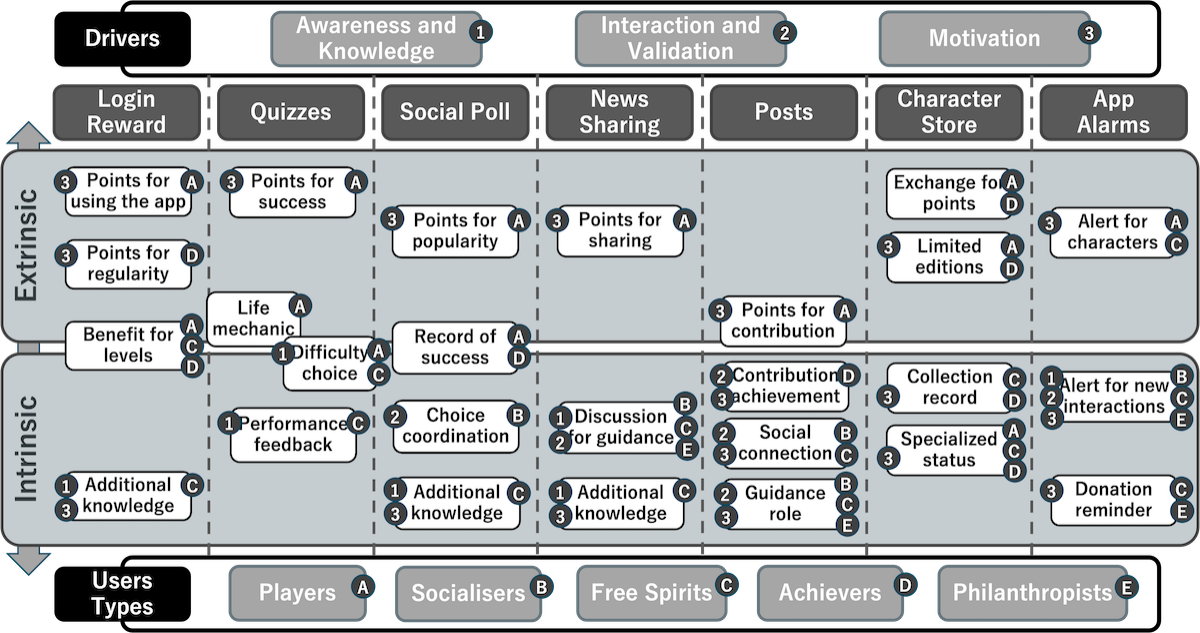
Outline of connection between drivers, users, features, and gamification elements.

Some of the mentioned intended connections between the drivers, the users, the features, and their gamification elements can be seen in Figure 1, which provides a more general outline of what we aimed to integrate as part of the activity loops.

#### Deploy of the Concept

**Figure 2:**
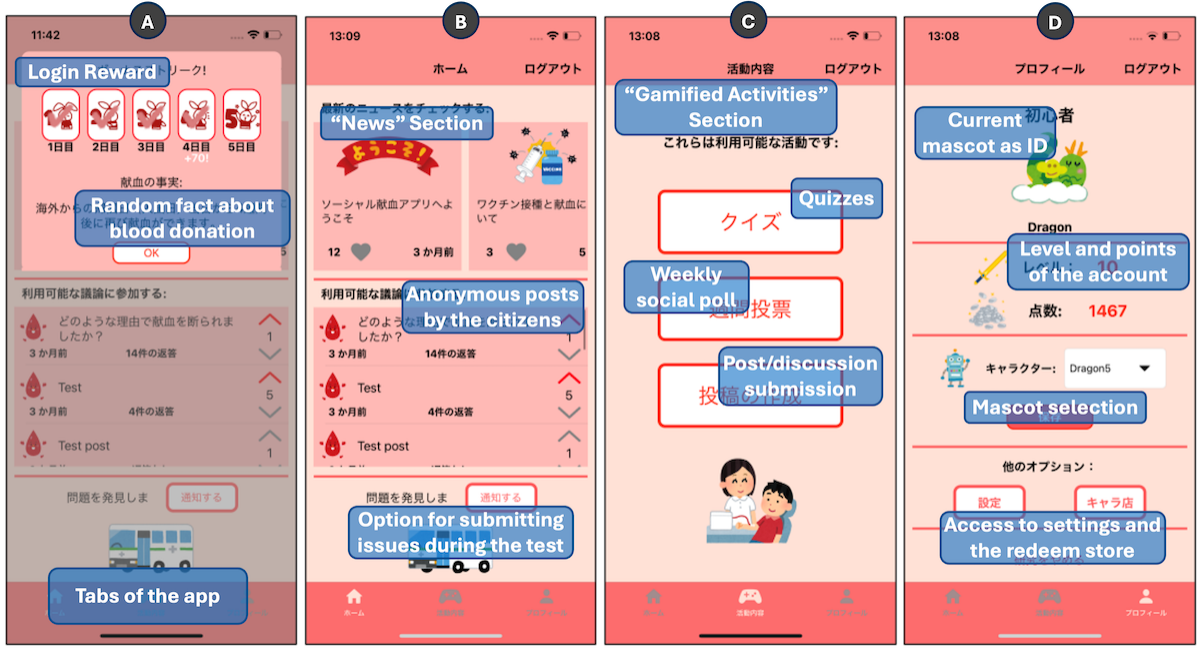
Screenshots of the main sections (Japanese version) of the prototype: (A) Home tab with the "Login Reward" as a pop-up; (B) Home tab screen; (C) Activity tab screen; and (D) Profile tab screen.

The proposed design was implemented in a basic mobile prototype, named as “Social Blood” app, to encapsulate the idea of a more interactive role from the citizens in blood donation. For the icon and the other illustrations of the prototype, public domain images were selected from the Japanese web page Irasutoya [51] for the test deployment. The main screens of the app are displayed in Figure 2.

A welcome screen was created for user registration with either an email or Facebook account. An additional functionality (In-app Survey) not related to the design was included for data collection. The app would check for surveys requested by the researcher or the staff and ask the user to answer them. Once the pending surveys were completed, the user was redirected to the main part of the app. If the user was accessing the app for the first time in the day, the “Login Reward” feature was shown to them.

A “Home” tab was created as the main interface available to the user. This section included features related to both learning and interaction, such as “News” and “Posts.” The user could check the number of likes and votes of any entry on this screen. An additional option not related to the design, “Error Report,” was also considered in this screen, to let users notify the administrators if any issue was found during the use of the app. An “Activity” tab was created to include the features that support learning. Users can access the “Quiz” and “Social Poll” features on this screen. Users could create a discussion post on this screen. Finally, a “Profile” tab was also created to show to the user information regarding the gamified elements of the app. This section of the app connects to the “Character Store” feature and to the “Settings Screen” screen, which includes the “Application Alarms” feature.

### Recruitment of Participants

To collect initial feedback regarding the prototype for its usability and acceptance by prospective users, a survey was performed with volunteers recruited on social networks. A digital flyer was posted with details of contact for the interested parties (Multimedia Appendix 1). Prospective participants were required to have an iPhone (Apple Inc.), be between 20 and 50 years old, and live in Japan for at least the last two months. Participants were recruited from June 17 to July 2, 2021. No incentives were used for the recruitment. Interested citizens received a Google Form (Google LLC/Alphabet Inc.) with the informed consent details and registration (Multimedia Appendix 2). If they signed up for participation, they later received an email with the following: a link to download the app, the user manual of the app (Multimedia Appendix 3), a list of main tasks to complete inside the app (Multimedia Appendix 4), and another Google Form link that contained an anonymous survey (Multimedia Appendix 5). Participants were asked to first download the TestFlight app from the Apple Store, and from there, install and use the approved version of the research prototype for a few days. They could then follow the tasks and complete the anonymous survey either through the app or through the Google Form once they deemed their test as completed. They could test the app and submit their answers to the survey until July 11, 2021.

### Ethical Considerations

The study focused on collecting preliminary feedback (usability and acceptance), so no sensible information was stored, and no risk nor effect was involved for the participants. With those points in consideration, considering the guidelines of the Kyoto University Graduate School and Faculty of Medicine Ethics Committee, it was not required to apply for ethical approval.

### Evaluation Details and Data Collected

Participants were instructed to attempt to complete the list of primary tasks outlined in the prototype app (Multimedia Appendix 4). Hereafter, these tasks are referred to as follows: Register and log-in (1. Log-in), fill out the survey (2. Survey), interact with news posts (3. News), interact in the discussion posts (4. Discussion), participate in the quiz activity (5. Quiz), participate in the weekly poll activity (6. Poll), submit a simple post (7. Post), acquire a new character (8. Buy), upgrade a new character in the Character Store (9. Evolve), select a new character for your profile (10. Select), and finalize their session (11. Log out).

In the Google Form, participants were asked to answer the following sections: demographic questions (age and gender), difficulty of task completion (questions about the previously mentioned list of tasks), System Usability Scale (SUS), and follow-up questions divided into acceptance questions (a Likert scale of 5 items) and opinion questions (free-text answers). Further details of these questions are provided in Multimedia Appendix 5. Additionally, participants were given a contact email to ask for support in case they had issues during the testing.

### Data Analysis

Statistics of mean, SD (*σ*), and standard error of the mean (*σ_M_*) were calculated for the average of task difficulty and the final SUS results. The SUS score per participant was calculated according to the standard assignment of points per type of question, which included positive and negative values [52,53].

Qualitative answers were grouped and summarized (if possible) following a simple semantic approach: we grouped the answers for each question and summarized the main ideas according to positive or negative feedback regarding the topic of the question. For the questions regarding the status of the app, we used the labels interface, functionality, and gamification to group the answers. The same categorization was followed for the questions regarding suggestions and improvements. Additional comments were not segregated but were individually considered and described, provided they were not redundant.

The analysis of the questions was carried out after the submission deadline for participant results had elapsed. Only submissions that were completed and received before the deadline were taken into account for the final analysis.

## Results

### Overview

From May 17 to July 2, 15 participants were recruited for the preliminary evaluation. A total of 13 participants created an account for the app and 11 participants submitted the final form. The full content of the answers is available in Multimedia Appendix 6. Participants ranged in age from 20 to 34 years. There were 10 male and 1 female participants. Descriptive statistics were used regarding the demographic variables of the participants.

### Difficulty of Task Completion

**Figure 3:**
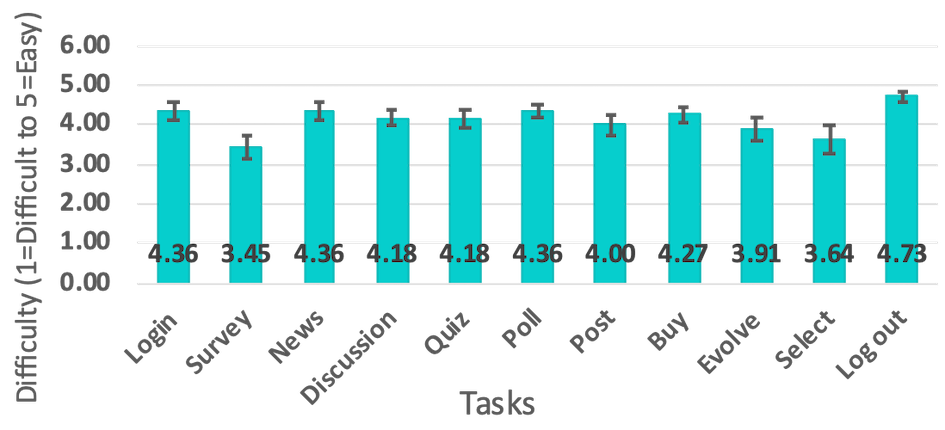
Subjective results of difficulty of each task.

Only 4 participants asked for support during the period of the evaluation. Questions were related to tasks 2, 7, 8, 9, and 10. Only tasks 9, 10, and 11 had 1 case each of not being completed. Regarding the difficulty level of each task, the results indicated that all of them were relatively easy to complete (4.13 average). The tasks that were considered the most difficult were task 2 (Completing the in-app surveys) and task 10 (Selecting a new character), as shown in Figure 3.

### System Usability Score

The SUS evaluation of our proposed app, as seen in Multimedia Appendix 7, showed a final score of 70.91 (scale 0-100, with 100 being the best usability), slightly above the average SUS score of 68 (C grade, percentile range of 41-59). The highest SUS score received by participants was 95, while the lowest score was 30.

Regarding the score per question, item 1 (“I think that I would like to use this application frequently”) showed the average lowest score from all the lists, with a value of 3.18. The highest score was for items 3 (“I thought the application was easy to use”) and 7 (“I would imagine that most people would learn to use this application very quickly”), with a value of 4.00.

### Follow-Up Questions

#### Acceptance and Qualitative Questions

Regarding the acceptance questions, participants responded positively to the app, expressing interest in its concept (3.82), likelihood to download it (3.55), and likelihood to recommend it to others (3.64).

For the qualitative questions, we summarized the answers for the main topics of the survey.

#### Goals of the App

Most participants considered a blood donation app concept useful or helpful. From them, 2 participants highlighted the possible impact of the deferral experience. The other 2 participants focused on the service being an app as a core value of the project.

#### Factors That Could Motivate Usage

Three participants emphasized that being aware of how they can contribute can help maintain their motivation; 3 participants mentioned interaction with others and popularity of the app as their motivation; 3 participants focused on the gamification aspects as one of their factors; 2 participants highlighted the social components as their drivers; and 2 participants indicated possible personal benefits for motivation.

#### Preferences About the App

Three participants liked the interactive possibilities of the app; 2 participants indicated the Quiz as their preferred feature; 3 indicated sharing and discussing as their favorite activities; 2 mentioned liking the activities involving characters; 1 participant indicated to like the interaction in general; and 1 participant indicated that they liked the aesthetic of the app the most.

#### Weaknesses of the App

Some participants recommended support of more languages so more citizens could benefit from the app. One participant indicated that the Quiz activity required improvement but did not specify reasons. Two participants indicated that the character functionality could be improved. One participant complained about the compulsory survey in the app because of its duration. One participant felt that not all the gamification features were connecting well with the goal of the project.

#### Current Status of the App

Only 1 participant mentioned that the current features might not be sufficient to support the goals of the app. They mentioned that while the app can be used to support deferred donors, it might not motivate them to promote blood donation. The other participants provided feedback regarding adjustments or fixes for the current version of the app (Textbox 1).

**Feedback regarding adjustments or fixes for the current version of the app.**

1. Regarding the User Interface
  Possibly change the color palette of the app or allow theme selection, as the red color might create discomfort. Implement support for the “Dark” mode, as it created issues with the color of text in the News and Post features.
2. Regarding Functionality
  Reduce the length of the In-App Survey. If possible, implement support for more languages, as it could help international students who want to donate.
3. Regarding Gamification
  Adjust the point requirement for characters, as it was too high in the test. Move the character selection option to a grid, so users can look at their whole collection when choosing. Add feedback messages in the Quiz about the points acquired.

#### Improvements and Suggestions

Participants were also asked about what they wanted to see for implementation in the future (Textbox 2).

**Participants’ expectations for implementation.**

1. Regarding Functionality
  Consider the inclusion of a feature to find locations for blood donations. Consider the inclusion of features to share the news and discussions on social networks. Allow to link or upload videos in the comments.
2. Regarding Gamification
  Consider adding the creation of groups or friend requests. Consider adding a “Gacha” option to acquire exclusive characters. Consider adding a ranking or certificate, similar to what is implemented in “Duolingo” [54].

#### Additional Comments

Some concerns about the information allowed in the Post feature were mentioned, as it could be nonrelated or harmful to the users. The usefulness of the app would be higher if medical institutions could provide information within it. It was suggested to highlight to the users the core goal of the app during the registration. It was also suggested to allow donors to know when their blood is used, as it could help to motivate them to continue to donate blood.

## Discussion

### Principal Findings

Current ICT services in blood donation aim to improve the citizens’ experience but do not focus on the deferral experience and its effects on prospective donors. This paper contributes to the field by debating the viability of implementing a system focusing on deferral and proposing a novel design to expand the concept of contribution and identity in blood donation. Our study indicates a missed opportunity in current services related to deferral. Potential users seem interested in an app supporting them in this area, and social gamification could make the role of a blood donor more approachable. However, our results, although slightly positive, require further validation due to the limitations, leaving room for discussion regarding the gamified design and the implemented prototype.

### Proposed Gamified Approach Reception and Shortcomings

- RQ1. Will our gamified design that focuses on the previously mentioned drivers with regard to the deferral experience in blood donation have a positive reception from potential users?

Participants’ favorable responses (average Likert scale score of 3.82) and positive opinions about the proposed functionality gave us an initial indication that the proposed project could be beneficial for the community. These results seem to align with ideas and concepts previously discussed in other studies. Previous studies discussed the relationship between knowledge of blood donation and intention to donate blood. However, only a couple of reviewed studies evaluated the ratio of knowledge regarding deferral. From our preliminary survey in Japan [49], 33% of nondonors did not know about the concept of deferral, with an additional 11% also unaware of the concept. Similar results were shown in [55], in which 90% of the participants never heard about the “donor deferral” term. This unawareness regarding deferral could be related to the positive response from the participants in our project, as either it introduced them to a new but relevant concept or it emerged as a service that could be valuable because of the low level of current support, which can be considered from their answers in the open questions. Furthermore, as participants expressed their positive intention to download the app (3.55) and to recommend it to others (3.64), the results suggest that there could not only be an interest but also an emerged necessity that has not appeared before because of the lack of awareness.

However, the current data are insufficient to reach a proper conclusion about the project acceptance, not only because of the small sample but also because of the scope of the participants, as it is not a proper representation of the target population. Additionally, the positive reception from the users could have been influenced by the Hawthorne effect [56], as the participants were aware of being part of an experiment, and the topic was related to a social contribution project. In this regard, a higher-scale study is required for further validation and analysis to decrease the effects of noise in the data and allow for more significant results.

Regarding the gamification aspect and its value, while the mentioned results were positive, their approval could have been related more to the goal of providing support. We delved into the comments of the participants about the design itself for possible conclusions. When asked about motivation to use the app and its best feature, some participants did indicate that the gamified aspects caught their interest and could even be driving motivators, highlighting the characters as part of it. These answers seem to suggest that the gamified components can play a role in, at least, capturing the interest of potential users. However, more detailed data are required to determine how beneficial is the integration of the gamified concepts in our proposed project. For example, asking participants for specific reasons why a gamification feature seems motivating to them or why it might feel discouraging. Besides, an additional evaluation regarding the impact is considered, as the value of the proposal can be confirmed if a positive effect can be determined. Comparing which features have more or less effect could also be important, as it could allow for the identification of factors to consider for future ICT-gamified implementations in blood donation.

Currently, while we discussed the importance of integrating the type of users, their motivations, and the MDA elements to nurture the drivers of interest, we have no specific data to indicate if our design has the desired effect or not. The data limitation becomes important with our goal of nurturing the intrinsic motivation of the citizens (prospective users), as we cannot recognize if the potential interest is related to the components that nurture the intrinsic motivation or the ones that do so for the extrinsic motivation. Analyzing some of the comments, most of the positive focus was on the *quiz* and the *characters*. These features, although designed considering an activity loop that could nurture intrinsic motivation, might not reach that goal in their current state. This weakness appears to be echoed in the feedback from 1 participant, who expressed dissatisfaction with the current state of the app, feeling that it falls short of achieving our design goals and lacks sufficient integration of features. As some participants showed interest in the social activities of the design (which are more related to intrinsic motivation), it might be worth it to redesign the current gamified activities to incorporate and integrate social components as part of the progress of the users.

We previously mentioned that some restrictions should be considered in a system related to blood donation, as some interactions could clash with the altruistic nature behind the donation act. To address that complexity, having a deeper understanding of game design itself is required. Learning from different and successful implementations of player interactions in game environments can lead us to a design that can properly nurture prospective blood donors’ social motivation. From the case studies in *The Gamification of Learning and Instruction Fieldbook* [57], an interesting idea is the implementation of specific types of leaderboards that encourage various forms of participation, thereby creating a stronger activity loop. Building on that concept, although we aim to steer clear of incentivizing competition in donation participation, we could adapt similar interactive mechanisms to enhance engagement in learning activities. For instance, in the Quiz activity, introducing a monthly leaderboard alongside corresponding achievements could offer users more personalized motivation compared with simply rewarding points. Another intriguing option could involve allowing users to accumulate questions they have answered correctly, which they could then use in a soft-competition interaction. In this scenario, users could anonymously challenge others using their question collections until their opponent provides an incorrect answer. With this revised structure, points serve as the initial incentive to engage with the Quiz feature. However, the interaction with others serves as an intrinsic motivator, encouraging users to strive for higher-difficulty questions to challenge others. Additionally, users may be motivated to continue learning or recalling information to avoid losing in these interactions.

Applying a similar rework infused with deeper game design insights could greatly enhance the experience for prospective users. However, before this step, gathering additional data on the project’s reception and soliciting input from more citizens would be invaluable. This information will help define the direction for implementing gamification strategies to encourage blood donation participation.

### Prototype Implementation Usability and Usefulness

- RQ2. Will our initial prototype implementation of the design be considered usable and useful in its current iteration?

Based on the initial average SUS score of 70.91, it seems that our proposed implementation is progressing in the right direction in terms of usability. Additionally, participants did not report significant issues regarding how to use the main options of the app, as they rated the difficulty level closer to “Somewhat Easy.” However, similar to the reception, we cannot draw definitive conclusions due to the small sample size and the potential influence of the Hawthorne effect. Moreover, the scores may have been positively biased due to the presence of an instruction manual and the support provided. Taking these factors into account, we directed our attention to the individual responses for more in-depth discussion.

Regarding difficulty, the activities with lower scores were those related to managing the characters (*evolving and selecting*), as well as the added survey functionality. From the comments, it appears that the functionality for upgrading the characters to their additional forms is not intuitive. The issue may stem from the fact that the options are spread across different screens, making it challenging to locate and connect them. Consolidating all the actions related to character management onto a single screen, separate from the character acquisition process, could potentially make the interface easier to use. In the case of the survey, the only complaint received was regarding its length, with participants finding it too long to complete. However, it received the lowest rating among the activities, suggesting that other participants may have also encountered issues with it. We can hypothesize that, aside from the length of the activity itself, participants may have been dissatisfied with its mandatory status rather than being optional. We could enhance the data collection process within the app by integrating it with gamification concepts, offering initial extrinsic rewards to users interested in participating. Ideally, we should also establish a loop that fosters participation through intrinsic motivation. This could involve designing activities or incentives that align with users’ intrinsic interests, values, or desires for personal growth or contribution. Furthermore, from specific results of the SUS score, the participant who gave the lowest score (30) cited issues with the user interface. Taking this into consideration, future implementations of the proposed design should allocate adequate time for interface functionality and compatibility tests.

Another point for analysis from the SUS results is the average score assigned to item 1 (“I think that I would like to use this application frequently”) of the survey. Although participants expressed positive sentiments regarding downloading the app and recommending it to others, the responses to item 1 indicated a nearly neutral position regarding the desire to use the app frequently. Indeed, the variation in results could stem from differing perspectives among user types. Nondonors might not use the app as frequently, even if they appreciate its concept. Similarly, donors with no deferral experience might use it for reference purposes, but perhaps not as frequently as deferred donors. Another possible and simple reason could be that participants might have had different interpretations of the term “frequently.” Besides the inclusion of the “user category” variable, future survey evaluations could use a support question to help identify the regularity (if either daily, weekly, or monthly, as examples) of usage of our proposed implementation.

Further data collection is still required to obtain more detailed feedback about the current implementation, as there might be additional issues or shortcomings from the usability or the usefulness that were not captured because of the small number of participants.

### Limitations

The study has multiple limitations that affect the reliability and generalization of the results. The small sample size of only 11 participants from Japan limits our ability to capture the true opinions of the various groups within the target population (prospective blood donors). Nondonors, donors, and deferred donors could have different perspectives and specific improvements regarding the design. Besides, although the recruitment was performed with Japanese material, we cannot confirm that only Japanese citizens participated in the evaluation. We have to consider that, although blood donation is seen as an altruistic and social activity in general, there can be differences in how individual values contribute to society according to one’s cultural background.

Recruiting participants through social networks and including ownership of an iPhone as part of the criteria may have biased the sample toward individuals with higher levels of technological literacy, potentially influencing the results of the SUS score. However, to mitigate this bias, consistent guidance materials and tasks were provided to ensure a similar starting point for all participants.

The final version of the prototype for evaluation was created within a limited timeframe and programmed solely by 1 (REC) researcher. This constraint impacted the resources available for implementing content and graphical user interface options. The workforce constraints also impacted the choice of the target system for development, leading to the selection of iOS for release due to the developer’s familiarity with it. Additionally, while the introduction of elements and activities involving user donation was considered, the acquisition and integration of these data into the current iteration of the project proved infeasible due to limitations related to permissions, partnerships, and time constraints.

Not collecting quantitative results regarding the value of the gamification aspect of the proposed design represents a significant weakness in the evaluation. Furthermore, the anonymous nature of the responses prevented the possibility of soliciting more detailed explanations regarding certain qualitative answers or comments from participants about the gamified components of the app. Indeed, it is crucial to address these shortcomings in future evaluations. Planning for a recruitment process that ensures a sufficient number of participants and obtaining ethical approval are essential steps for conducting a more comprehensive evaluation.

### Conclusions

ICT systems have gained significant recognition and reliability across various fields, including within the realm of blood donation. We sought to explore previous work related to deferred donors and identify areas for further improvement. In addition to providing automated services, certain ICT projects have prioritized enhancing user motivation by incorporating gamification into their design. However, upon reviewing the current literature, it became apparent that only a few, if any, of the existing systems have specifically addressed the experience of deferral or its implications. In this research, we introduced an innovative ICT gamified design and implementation aimed at addressing this overlooked issue. Additionally, we offered an initial assessment of the project’s potential reception, usability, and usefulness. Further enhancements can be made to the design of activities, which currently rely primarily on extrinsic motivation elements, to incorporate more social interaction. This would create an enriched activity loop that fosters intrinsic motivation. Further research could involve a more specialized and longitudinal design evaluation with a larger sample size. Understanding which specific features or gamification elements influence citizens’ intentions or behaviors regarding their role in blood donation could be crucial for future design endeavors. Moreover, it could serve as a reference point for official ICT implementations in blood donation services.

## Appendix

Multimedia Appendix 1 Study recruitment flyer—Japanese version.

Multimedia Appendix 2 Informed consent—Google Form sections for recruitment.

Multimedia Appendix 3 App user manual—English version (from right to left).

Multimedia Appendix 4 List of tasks for the app evaluation.

Multimedia Appendix 5 Study main survey—acceptance, System Usability Scale (SUS), and feedback.

Multimedia Appendix 6 Study survey data—formatted, anonymized, and translated to English.

Multimedia Appendix 7 System Usability Scale (SUS) results from each user with average and SD.

## Abbreviations

ICT: information and communications technology
RQ: research question
SDT: Self-Determination Theory
SUS: System Usability Scale
TPB: Theory of Planned Behavior
