# Supplementary figures and images for: Gamification Approach to Provide Support About the Deferral Experience in Blood Donation: Design and Feasibility Study
Source: JMIR Hum Factors. 2024 Jun 14;11:e50086. doi: 10.2196/50086 (PMC11214031; doi:10.2196/50086)

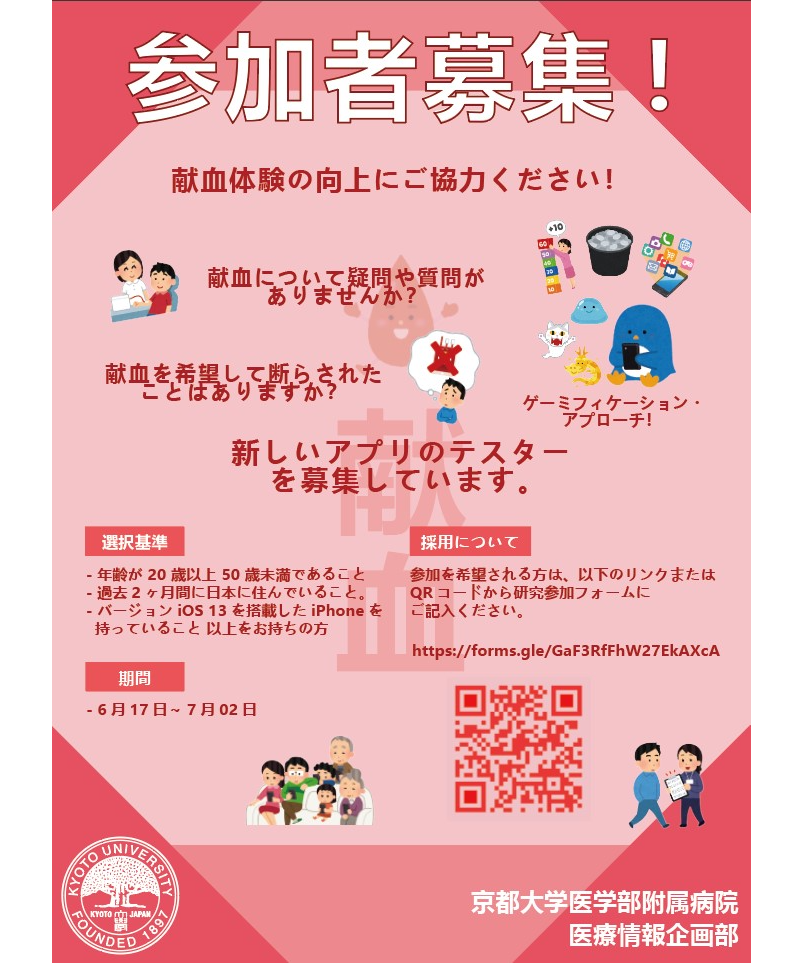

Supplement: Multimedia Appendix 1 [file humanfactors_v11i1e50086_app1.png]

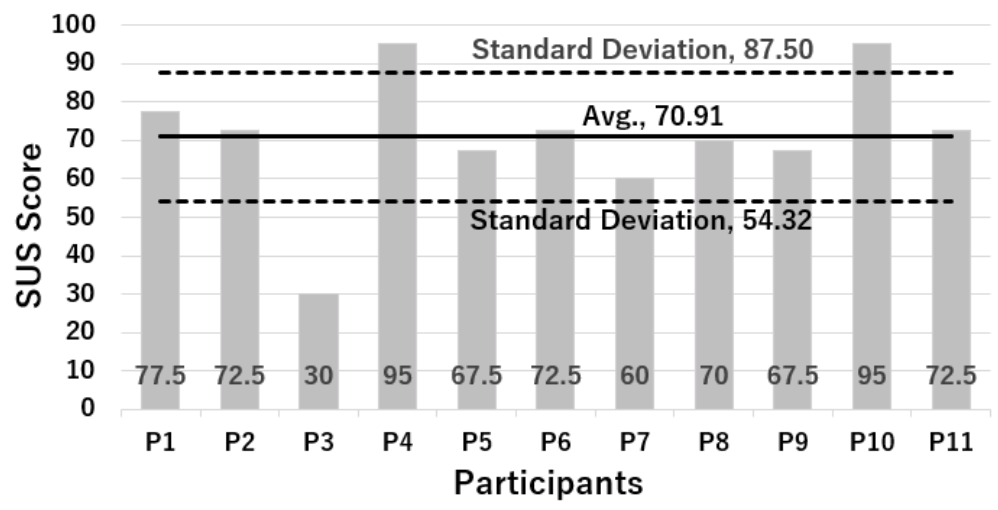

Supplement: Multimedia Appendix 7 [file humanfactors_v11i1e50086_app7.png]
