# Supplementary material for: Gamification Approach to Provide Support About the Deferral Experience in Blood Donation: Design and Feasibility Study
Source: JMIR Hum Factors. 2024 Jun 14;11:e50086. doi: 10.2196/50086 (PMC11214031; doi:10.2196/50086)
Supplement: Multimedia Appendix 2 [file humanfactors_v11i1e50086_app2.docx]

# Multimedia Appendix 2

This is a Multimedia Appendix to a full manuscript published in the J Med Internet Res. For full copyright and citation information see <http://dx.doi.org/10.2196/jmir.50086>.

The Appendix consist of the detailed list of sections included in the Google Form provided to the citizens that were interested in participating in the study.

## Informed Consent – Enrolling

The Google Form provided was used to deliver the Informed Consent, the enrollment and the final evaluation questions. The Google Form included 4 sections: Introductory Message; Explanation about the evaluation; Registration Form; and Completion of the Form. Below we indicate the details about each section.

### Introductory Message Form

Participants recruitment: Pilot test of a gamified mobile application aims to increase deferred donors' intention to participate in blood donation.

Message to the Participants: Dear Volunteers, first of all, thank you very much for your cooperation and interest in this research. This evaluation is part of the research I am conducting as a Master's (M2) student in the Department of Medical Informatics, Division of Social Informatics, Graduate School of Informatics, Kyoto University. Our team is working on a research project that aims to use a gamified mobile application to improve the willingness of blood donors who have been rejected once to donate blood again. At this stage, we would like to conduct a pilot test on the usability and acceptability of the application.

### Explanation of the evaluation

Purpose of the Study: The purpose of this study is to evaluate the acceptability and usefulness of mobile application games and to collect opinions from participants.

Method and Duration of the Study: This research is being conducted by Roberto Espinoza Chamorro, a master's student (M2) at the Department of Medical Informatics, Division of Social Informatics, Graduate School of Informatics, Kyoto University. In this assignment, participants are given access to a test version of a game application and are asked to test its functionality. After the participants have finished testing the app, they will be asked to fill out a questionnaire (Google Form).

The primary assessment items consist of the first three sections of the questionnaire, which are Likert scale questions. The secondary evaluation items consist of free-text comments from the participants at the end of the questionnaire.

Expected benefits: Participants will not receive any direct benefits or rewards for participating in this study, but the ripple effect of the study may positively impact the process of blood donation and benefit society.

Possible disadvantages: Participants will be required to download a game app. Using the application also requires access to the Internet, which may incur data usage charges. It will also take about 20-30 minutes to test the application and another 20-30 minutes to complete the final survey. No physical or mental harm is expected from participating in this study.

Inclusion criteria:

- Age between 18 and 50 years.
- Having lived in Japan for the past 2 months.
- Owning an Apple iPhone 8 or above, with operating system updated to iOS 13 or above.

Exclusion criteria:

- Participants who, due any condition, are not capable of giving their own consent to participate in this research.
- Participants who are not able to read or understand Japanese.

Privacy Protection: Participant’s information and application usage data collected during the tests will be permanently destroyed after the evaluation period is finished. The personal data informed in order to provide the application for participants (e-mail) will be used for this purpose only, and will not be collected as part of this research, nor shared with any third parties. The data collected in this research does not include any private information: only the answers to the final questionnaire will be stored and analyzed, in anonymous form.

Funding of the Research: This research is funded by Kyoto University Hospital’s operational expenses budget.

Conflicts of Interest: There is no conflict of interest to be declared.

Handling of Information after Completion of the Research: Data collected during the study (answers to the final questionnaire) will be stored in anonymous form only.

Statistics and summarized versions of this data may be included in publications such as academic journals, conference proceedings, and others. Following the Kyoto University Hospital ethical regulations, the anonymous data will be kept securely in a password protected server for 10 years and safely discarded after this period.

Name of the Research Organization: Kyoto University, Medical Informatics Laboratory

Contact for Inquiry: Patient Consultation and Support Center, Kyoto University Hospital . Tel: 075-751-4748. E-mail: ctsodan@kuhp.kyoto-u.ac.jp

Consent Form:

- Question: Do you voluntarily agree to participate in this study?
- Description: We received a full written explanation of the research ``Pilot test of a gamified mobile application aims to increase deferred donors' intention to participate in blood donation''
- Answer 1: I understand the contents of this study and agree to participate in the
- Answer 2: I prefer to not participate in this study.

### Registration Form

In order to test this app, you will first need to download the TestFlight Application from the App Store and let us add you to the test group. Please provide us with the following information that we need to add you to the test group.

- Name (used only to properly send the email with the next steps of the study)
- E-mail (used only to register the participant in the App Store Connect group)

### Completion of the Form

Thank you for consenting to participate in this study. We will contact you by e-mail as soon as possible. The message will contain two sections.

- A link and QR code to download the application by TestFlight (Fig. 1).
- A link and QR code to access the final survey form.

Additionally, a PDF file of the user manual will also be attached to the email.


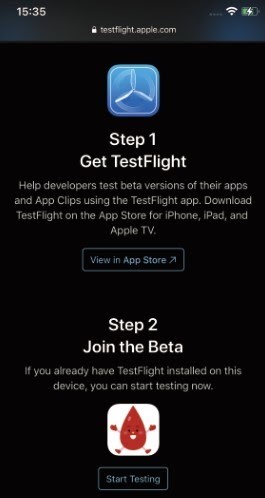


Figure 1. Screen of the download instructions via TestFlight.

After you have tested the app, please complete the provided Google Form questionnaire. We appreciate your cooperation in this study.
