# Supplementary material for: Gamification Approach to Provide Support About the Deferral Experience in Blood Donation: Design and Feasibility Study
Source: JMIR Hum Factors. 2024 Jun 14;11:e50086. doi: 10.2196/50086 (PMC11214031; doi:10.2196/50086)

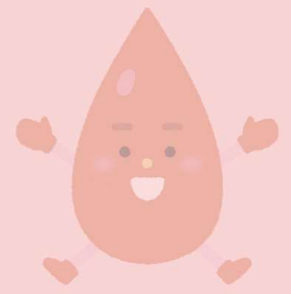

献血

Social Blood App
使用方法

medical informatics

Support Information

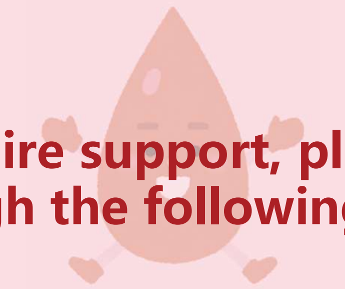

If you require support, please contact us through the following channels:

Telephone:

075-366-7701

Weekdays at 10 a.m - 12 p.m, 1 p.m - 4 p.m

E-mail:

medinfoq@kuhp.kyoto-u.ac.jp

https://www.kuhp.kyoto-u.ac.jp/~mi/

Contents

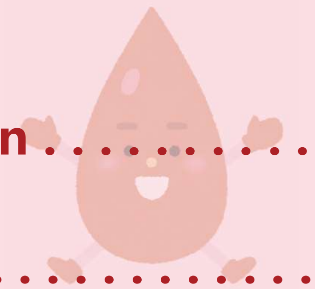

Registration 1

Surveys 2

Home Tab 3

Activities Tab 5

Profile Tab 8

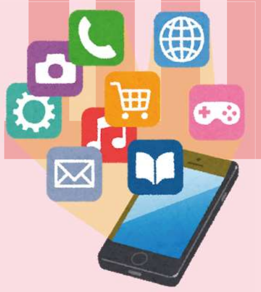

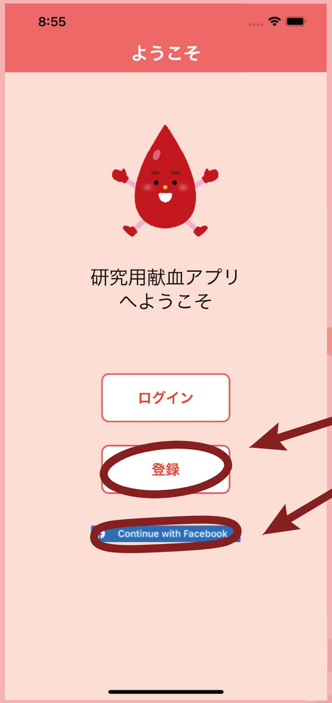

Registration

You can create an account with your mail or with an SNS account

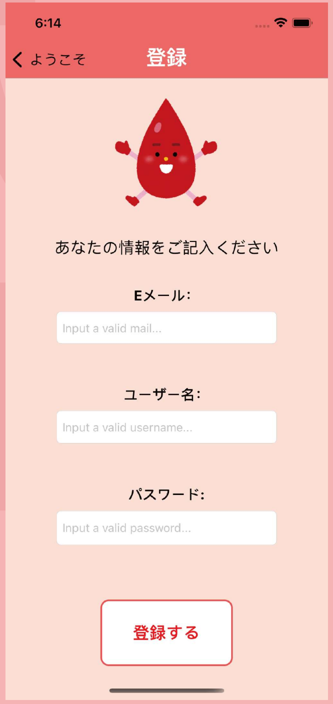

Mail registration requires authentication of the user.

Please click the validation link you will receive in your mail

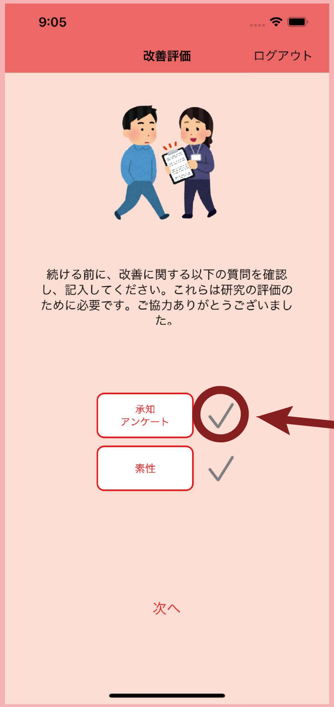

Surveys

For the first login, you will be required to complete some surveys

After completing a survey, the check will activate. All surveys have to be completed to continue

Surveys will take between 3 to 10 minutes

For this test, the information of the surveys and the informed consent will not be stored

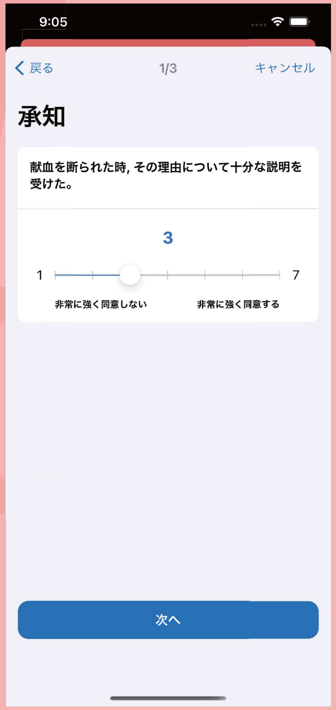

Home Tab

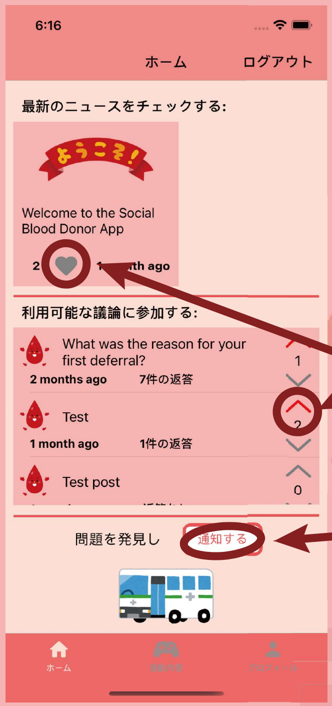

You can access different news and discussions related to blood donation.

You can like the news as well as upvote or downvote a discussion.

If something is out of place, or if you detect a misbehaviour, please let us know!

Login Reward:

You can earn points by login into the app once a day. The rewards reset every week.

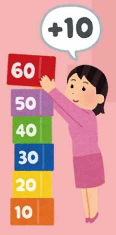

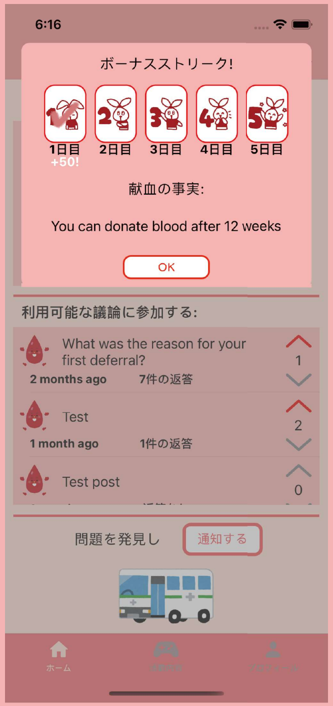

News Section:

Get news related to blood drives, current level of donations, blood campaigns, and information about how to act after a deferral.

Feel free to comment about the news, or to suggest about desired information for future posts.

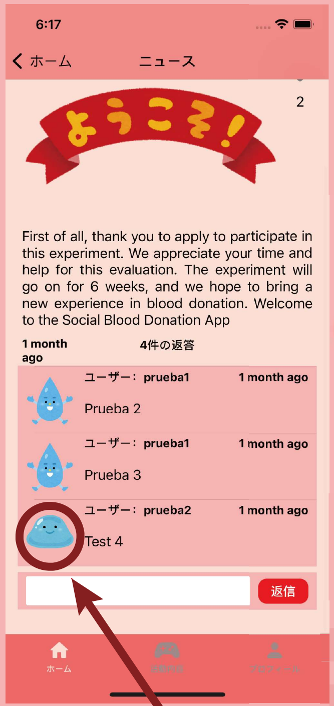

User posts will show their selected character!

Discussion Section:

Access to discussions created by the researchers or by other users.

The creator of the post will remain anonymous. Feel free to ask about blood donation and/or deferral worries or issues.

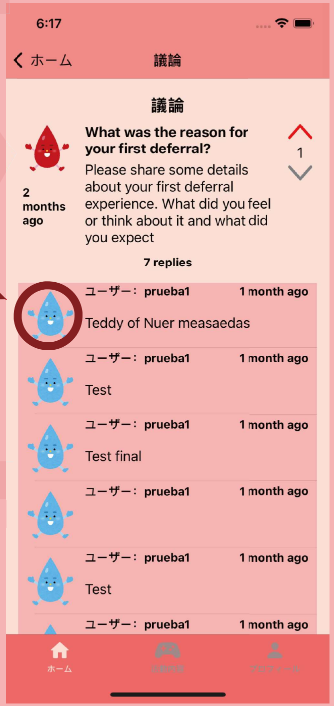

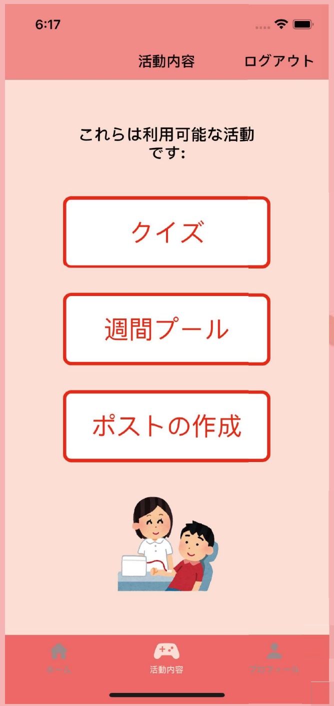

Activities Tab

Participate in quizzes or in the social pool to gain more points!

You can also submit a new discussion post from here

Post Section:

The title will be visible from the Home page.

Please try to give more details in the body section.

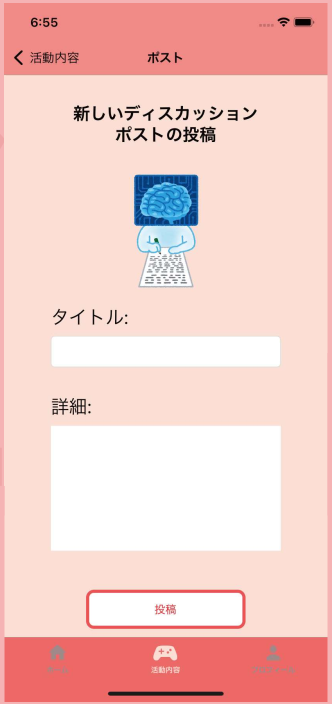

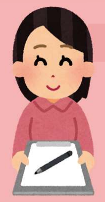

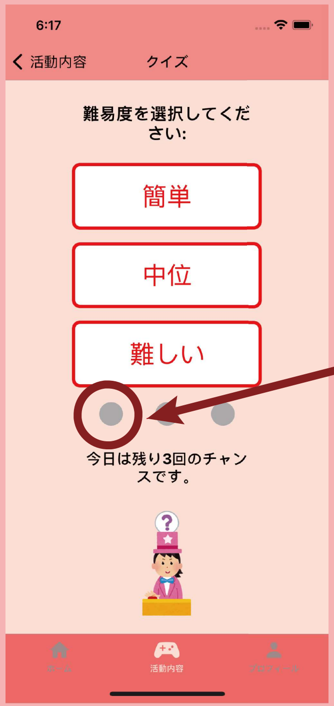

Quiz Section:

You can select between 3 types of difficulty. Harder questions give more points!

This part will show your results for each quiz.

You have 3 chances per day.

Everytime you select a level, you will get a random question.

This part will show the question

Click on the option to select your final answer

This part will show the answer

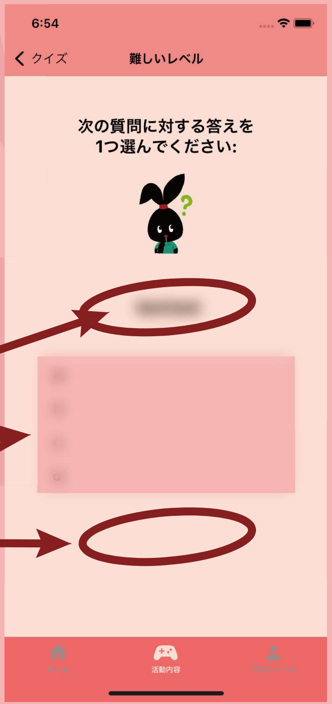

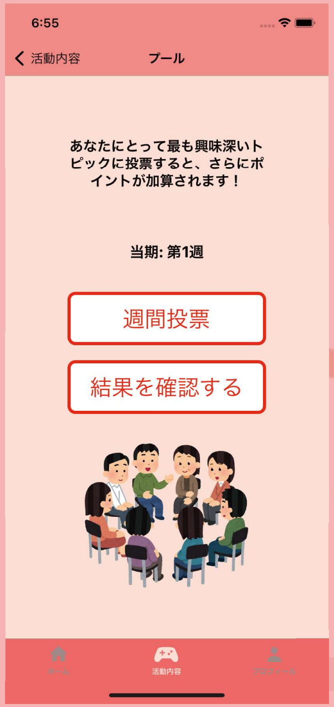

Poll Section:

Vote for the most interesting fact about deferral.

You can participate once a week! Every week, there will be a poll of new options.

You can redeem your points at the start of the new week.

Voting has a deadline for each week. Be sure to not miss it!

The options will be open for selection until the deadline.

Save your choice, and went for the later results!

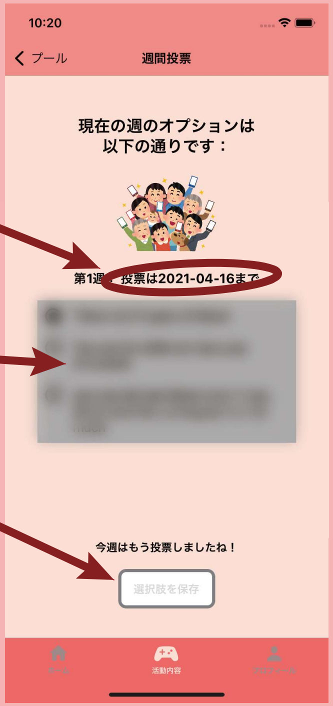

Profile Tab

You can update your preferences or access the character shop from this section.

Shows your current character

Level of your account

Remaining points

Level of your character

Type of your character

Select from your collected characters

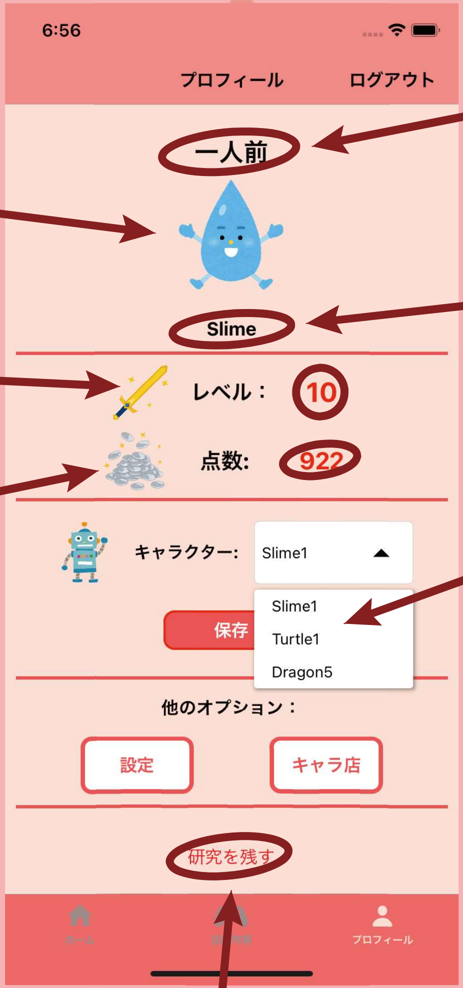

You can quit the research with this option. Your data will be disabled and not considered for the final evaluation of the reasearch.

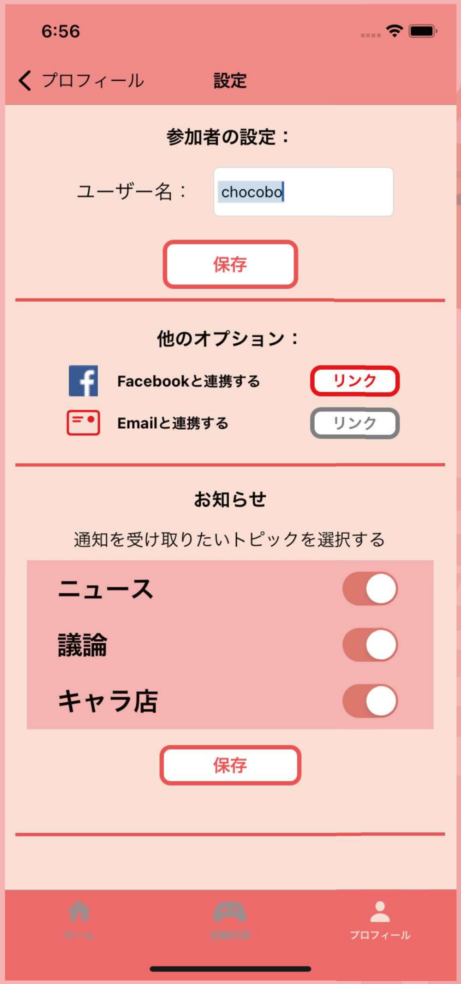

Settings Section:

Update your username. Please consider that it will be visible to other users.

Link SNS to your email account for easier access. SNS data will not be used expect for login to this app.

The app will alert you about new post done by the main researcher (news and discussions), as well as about new characters in the shop.

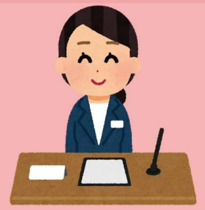

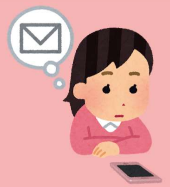

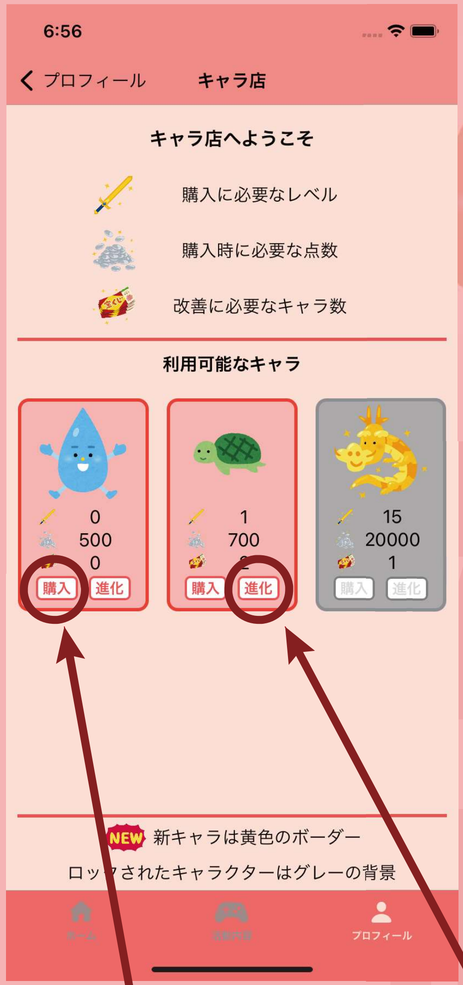

Shop Section:

Buy characters using the points you from your account.

Your character will be shown in the social activities!

Each character has up to 3 forms. Collect the same character to evolve it!

New characters will be added regularly.

Try and collect them all!

Evolve your character once you have enough copies!

Acquire the new characters!

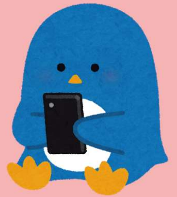

Supplement: Multimedia Appendix 3 [file humanfactors_v11i1e50086_app3.pdf]
