# Supplementary material for: Gamification Approach to Provide Support About the Deferral Experience in Blood Donation: Design and Feasibility Study
Source: JMIR Hum Factors. 2024 Jun 14;11:e50086. doi: 10.2196/50086 (PMC11214031; doi:10.2196/50086)
Supplement: Multimedia Appendix 4 [file humanfactors_v11i1e50086_app4.docx]

# Multimedia Appendix 4

This is a Multimedia Appendix to a full manuscript published in the J Med Internet Res. For full copyright and citation information see <http://dx.doi.org/10.2196/jmir.50086>.

The Appendix consist of the details of the main tasks of the activities expected to be completed in the prototype application. This list was included in the Google Form provided to the citizens.

## Tasks for Usability Evaluation

For this evaluation, we ask you to try to complete the following activities inside the application. You can refer to the attached Manuals in the e-mail for additional details of each of the sections and functionalities in the application.

Once you have finished your testing, you are free to interact with the application as you please. After that, please complete the survey previously provided in the e-mail. Thanks again for your cooperation.

- Getting Started Section
  1. Register and login: Create an account in the application and access it with the chosen credentials.
  2. Fill out the survey and the informed consent: Complete the automated survey sections inside the application. If accessing for the first time, the application will show a couple of surveys to complete, including a digital version of the informed consent.
- Home tab
  1. Comment and Like any News posts: The home tab includes a section with examples of News article. The user should interact with them as they deem possible, but at least with one of the available ones.
  2. Comment on and Upvote or Downvote in the Discussion posts: The home tab includes a section with examples of Discussion submissions. The user should interact with them as they deem possible, but at least with one of the available ones.
- Activity tab
  1. Participate in the quiz activity: Use the gamification section that consists of a deferral-related quiz activity with progressive difficulty selection.
  2. Participate in the weekly poll activity: Use the gamification section that consists of a deferral-related indirect social poll, with participation record. For this evaluation, the results will be provided every 2 days to allocate more points to the participants.
  3. Make a simple post: Submit your own content for interaction with other users. The option will be unlocked from the start for this evaluation.
- Profile Tab
  1. Acquire a new character: Spend you points collected from the other activities to exchange a new character in the Character Store.
  2. Upgrade (evolve) a new character: Spend you points collected from the other activities to upgrade one of your available characters to its next stage in the Character Store.
  3. Select a new character for your profile: Change your Character profile picture to a different one from the default option.
- Additional Activity
  1. Log out: Disconnect your session from the application through any of the available options.
