# Supplementary material for: Gamification Approach to Provide Support About the Deferral Experience in Blood Donation: Design and Feasibility Study
Source: JMIR Hum Factors. 2024 Jun 14;11:e50086. doi: 10.2196/50086 (PMC11214031; doi:10.2196/50086)
Supplement: Multimedia Appendix 5 [file humanfactors_v11i1e50086_app5.docx]

# Multimedia Appendix 5

This is a Multimedia Appendix to a full manuscript published in the J Med Internet Res. For full copyright and citation information see <http://dx.doi.org/10.2196/jmir.50086>.

The Appendix consist of the different sections of questions provided in the final Google Form. Each of the subsections was part of its own page in the form, as to not show all of the questions to the user at the same time. The survey did not collect the email, limited only 1 submission per participant, allowed for response editing, presented the questions in the same order for all participants, and showed their current progress of the survey.

## System Usability Questionnaire

### Demographic Questions

- Age
  (20-24/25-29/30-34/35-39/40-44/45-49)
- Gender
  (Male/Female/Other)

### Difficulty of Task Completion

- Were you able to complete Task 1 (Registration and Login)?
  (Yes/No)
- Please rate the difficulty of Task 1.
  (1. difficult, 2. somewhat difficult, 3. normal, 4. somewhat easy, 5. easy)
- Were you able to complete Task 2 (filling out the questionnaire in the application and giving informed consent)?
  (Yes/No)
- Please rate the difficulty of Task 2.
  (1. difficult, 2. somewhat difficult, 3. normal, 4. somewhat easy, 5. easy)
- Were you able to complete Task 3 (commenting and liking news postings)? (Yes/No)
- Please rate the difficulty of Task 3.
  (1. difficult, 2. somewhat difficult, 3. normal, 4. somewhat easy, 5. easy)
- Were you able to complete Task 4 (Comment and agree or disagree with the discussion poll)?
  (Yes/No)
- Please rate the difficulty of Task 4.
  (1. difficult, 2. somewhat difficult, 3. normal, 4. somewhat easy, 5. easy)
- Were you able to complete Task 5 (Participate in the quiz activity)?
  (Yes/No)
- Please rate the difficulty of Task 5.
  (1. difficult, 2. somewhat difficult, 3. normal, 4. somewhat easy, 5. easy)
- Were you able to complete Task 6 (Participate in the weekly voting activity)? (Yes/No)
- Please rate the difficulty of Task 6.
  (1. difficult, 2. somewhat difficult, 3. normal, 4. somewhat easy, 5. easy)
- Were you able to complete ask 7 (Make a simple post)?
  (Yes/No)
- Please rate the difficulty of Task 7.
  (1. difficult, 2. somewhat difficult, 3. normal, 4. somewhat easy, 5. easy)
- Were you able to complete Task 8 (Acquire a new character at the Character Store)?
  (Yes/No)
- Please rate the difficulty of Task 8.
  (1. difficult, 2. somewhat difficult, 3. normal, 4. somewhat easy, 5. easy)
- Were you able to complete Task 9 (go to the character store and evolve a new character)?
  (Yes/No)
- Please rate the difficulty of Task 9.
  (1. difficult, 2. somewhat difficult, 3. normal, 4. somewhat easy, 5. easy)
- Were you able to complete Task 10 (Select a new character and save changes)? (Yes/No)
- Please rate the difficulty of Task 10.
  (1. difficult, 2. somewhat difficult, 3. normal, 4. somewhat easy, 5. easy)
- Were you able to complete Task 11 (Logout)?
  (Yes/No)
- Please rate the difficulty of Task 11.
  (1. difficult, 2. somewhat difficult, 3. normal, 4. somewhat easy, 5. easy)

### System Usability Scale

Choose the answer according to your degree of agreement. (1) means that you “totally disagree” with the statement, and (5) means that you “highly agree” with the statement.

(1) Totally disagree.

(2) Disagree

(3) Neither agree nor disagree

(4) Agree with you

(5) Very much agree

There are no right or wrong answers to the following questions. Also, please feel free to answer these questions, as the purpose of this survey is to improve the application, not to evaluate your ability.

1. I think that I would like to use this application frequently.
2. I found the application unnecessarily complex.
3. I thought the application was easy to use.
4. I think that I would need the support of a technical person to be able to use this application.
5. I found the various functions in this application were well integrated.
6. I thought there was too much inconsistency in this application.
7. I would imagine that most people would learn to use this application very quickly.
8. I found the application very cumbersome to use.
9. I felt very confident using the application.
10. I needed to learn a lot of things before I could get going with this application.

### Follow-up Questions

Acceptance Section:

- I liked the concept of this application
  (1.Totally disagree, 2.Disagree, 3.Neither agree nor disagree, 4.Agree, 5.Very much agree).
- Assuming the application is free, how likely are you to download it?
  (1.Very unlikely, 2.Unlikely, 3.Don't know, 4.Likely, 5.Very likely)
- How likely are you to recommend this application to others?
  (1.Very unlikely, 2.Unlikely, 3.Don't know, 4.Likely, 5.Very likely)

Free-text Questions:

Please be as detailed as possible in your description. The questions from this section are optional. This section should take 8 to 12 minutes to complete.

1. The goal of this application is to improve the intention of participants to donate again after possible deferral case. For that, the application looks to give support about the deferral experience in blood donation with information and a channel for social discussion and/or interaction, integrated with Gamification to keep the participants’ motivation in the activities.
   1. Do you think the functions of this application are adequate?
   2. Are there any features in this application that should be improved or removed?
   3. Are there any functions that are lacking or that you would like to see added to this application?
2. What factors could motivate you to use the application?
3. What is the best feature you found in the app?
4. What features of the application have you found to be the least?
5. What aspects did you like the most about the application's goals?
6. Please, if you could give as additional comments and suggestions.
